# Clinical Features of Cluster Headache: A Hospital-Based Study in Taiwan

**DOI:** 10.3389/fneur.2021.636888

**Published:** 2021-04-07

**Authors:** Chien-An Ko, Guan-Yu Lin, Chi-Hsin Ting, Yueh-Feng Sung, Jiunn-Tay Lee, Chia-Kuang Tsai, Chia-Lin Tsai, Yu-Kai Lin, Tsung-Han Ho, Fu-Chi Yang

**Affiliations:** ^1^Department of Neurology, Tri-Service General Hospital, National Defense Medical Center, Taipei, Taiwan; ^2^Department of Neurology, Songshan Branch, Tri-Service General Hospital, National Defense Medical Center, Taipei, Taiwan; ^3^Department of Internal Medicine, Taichung Armed Forces General Hospital, Taichung, Taiwan

**Keywords:** cluster headache, headache, Taiwan, clinical features, cross-sectional study

## Abstract

Most previous studies on cluster headache (CH) focus on Western populations. This study aimed to investigate the clinical characteristics of CH in a neurology outpatient population in Taiwan. A cross-sectional survey was conducted from July 2015 to June 2019 in a medical college affiliated with a tertiary care hospital (Tri-Service General Hospital) in Taiwan. All consecutive patients reporting headache as their chief complaint were asked to participate in a face-to-face interview with a qualified headache specialist and to complete a detailed self-administered questionnaire. The diagnosis of CH was made according to the Third edition of the International Classification of Headache Disorders. The subjects comprised 80 consecutive new CH patients (13 women and 67 men; ratio, 1:5). The mean age at presentation was 36.0 ± 10.8 years (range, 16–64 years), mean age at onset was 27.2 ± 12.1 years (range, 5–65 years), and mean time lag before diagnosis was 9.3 ± 10.5 years (range, 0–46.4 years). Of the total CH patients, 25.3% reported feelings of restlessness during headache episodes. A seasonal predilection was reported by 18% of the CH patients. The use of tobacco was the most common (44/80 patients). Chronic CH was only observed in 5% of the patients and only one patient (1.3%) reported both a positive family history for CH and aura. Features of CH in Taiwanese patients differed from that of Caucasian patients; a lower prevalence of chronic CH, positive family history of CH, and occurrence of aura may be less common in the former than in the latter.

## Introduction

Cluster headache (CH) occurs in 0.1% of the general population (1). Characterized by episodes of excruciating unilateral headache or facial pain lasting 15–180 min, CH is the most common trigeminal autonomic cephalalgia and is considered the most severe primary headache disorder (2). Typically, CH attacks occur in association with ipsilateral autonomic symptoms such as ptosis and/or miosis, conjunctival injection and/or lacrimation, nasal congestion and/or rhinorrhea, forehead and facial sweating, and eyelid edema (2). Attacks can take place every 2 days up to eight times daily, with a propensity for nocturnal attacks (3). CH also exhibits a remarkable circadian and circannual rhythmicity, with attacks often occurring at the same time, each day, during episodes, and can be sustained for weeks or months (in-bout periods), separated by complete remission periods (out-of-bout periods) (4). Approximately 10–15% of patients with CH have the chronic form, in which remission periods are either absent or last <3 months, for at least 1 year (2, 5). Current neuroimaging studies support that in addition to the hypothalamus, pain-modulatory circuitry with dynamic transitioning between in- and out-of-bout periods are involved in the CH pathophysiology. Activation of the trigeminovascular system with consequent release of vasoactive neuropeptides plays a major role in CH pathophysiology as well. Nevertheless, data regarding CH in Eastern countries are scarce in the literature. This article aimed to survey the similarities and differences in the clinical features of CH among neurology outpatient populations in Taiwan and Western countries.

## Materials and Methods

### Patients

We conducted a hospital-based retrospective study at a medical college affiliated to a tertiary care hospital (Tri-Service General Hospital) in Taiwan, between July 2015 and June 2019. The study protocol was approved by the Tri-Service General Hospital Institutional Review Board (No. 2-106-05-163).

The study population consisted of patients who presented to the neurology unit and were diagnosed with CH, as defined by the International Classification of Headache Disorders, Third edition (2).

All the patients provided written informed consent before enrolment and underwent detailed clinical evaluation by a headache specialist. Clinical data collected included sex, age at onset, disease course, family history, history of smoking and drinking, location, quality, autonomic features, additional features, laterality, intensity, and duration. In addition to the routine blood tests, magnetic resonance imaging (MRI) of the brain was performed for all the patients. Brain MRI was carried out to exclude underlying structural lesions such as vascular malformations, neoplasms, and cervicocephalic arterial aneurysms/dissections that could mimic CH. After excluding the patients with secondary headache disorders, a total of 82 CH patients were included in this study. However, two patients were excluded due to incomplete response (Supplementary Figure 1); therefore, 80 patients with primary CH were included in the final analysis. The patients were also classified by CH subtype into either episodic or chronic CH groups.

### Statistics

We reported the patients' data using frequencies and percentages for categorical variables. Data were presented as mean and standard deviation (SD) for continuous variables.

## Results

We collected data of 80 CH patients; of these, 67 (83.8%) were male and 13 (16.2%) were female (male-to-female ratio, 5:1). Table 1 lists the demographic profile of the patients. The majority of the study patients had episodic CH (*n* = 76, 95%); only four (5%) had the chronic subtype. The mean age at presentation was 36.0 ± 10.8 years (range, 16–64 years), mean age at onset was 27.2 ± 12.1 years (range, 5–65 years), and mean time lag before diagnosis was 9.3 ± 10.5 years (range, 0–46.4 years). The latency period was 7.8 (*SD* = 8.1), 14.5 (*SD* = 9.6), 7.7 (*SD* = 12.2), 3.7 (*SD* = 3.8), and 12.5 (*SD* = 8.9) years in 2015, 2016, 2017, 2018, and 2019, respectively. The result of the linear trend was not significant (*P* = 0.896) (not shown). Only one (1.3%) patient reported a family history of CH. Forty-four patients (55.7%) smoked and 11 (14.1%) consumed alcohol (Table 1).

**Table 1:** Demographic profile (*N* = 80).

**Variable**	**Frequency (percentage) or mean ± SD**
**Type of cluster headache**	
Episodic	76 (95.0)
Chronic	4 (5.0)
Age at presentation in years	36.0 ± 10.8
Age at onset in years	27.2 ± 12.1
Family history	1 (1.3)
Latency in years	9.3 ± 10.5
Smoking	44 (55.7)
Alcohol	11 (14.1)

*SD, standard deviation*.

Table 2 lists the characteristics of pain experienced by the patients. Pain was localized predominantly over the retro-orbital (73.8%) and temporal regions (67.5%), followed by the forehead (42.5%) and occipital region (43.8%). The most common quality of pain was pulsating (44.3%), followed by exploding (30.4%) and stabbing (24.1%). The most frequent autonomic features were lacrimation (64.5%), followed by rhinorrhea (43.5%), conjunctival injection (32.3%), facial sweating (27.4%), restlessness (25.3%), nasal congestion (19.4%), blepharedema (19.4%), ptosis (14.5%), facial edema (11.3%), and miosis (4.8%). The additional features most reported were nausea (46.8%) and phonophobia (45.6%), followed by photophobia (44.3%) and vomiting (30.4%). Thirty-three (41.3%), 27 (33.8%), and 20 (25%) had right and left unilateral and side shift CH, respectively. Mean pain intensity was 7.5 ± 2.3 (range, 1–10), indicating a moderate to severe degree of pain. Only one patient (1.3%) reported experiencing visual aura before a CH attack.

**Table 2:** Characteristics of pain (*N* = 80).

**Variable**	**Frequency (percentage) or mean ± SD**
**Site of pain**	
Retro-orbital	59 (73.8)
Temporal	54 (67.5)
Forehead	34 (42.5)
Occipital	35 (43.8)
**Quality of pain**	
Pulsating	35 (44.3)
Stabbing	19 (24.1)
Explosive	24 (30.4)
**Autonomic features**	
Conjunctival injection	20 (32.3)
Lacrimation	40 (64.5)
Nasal congestion	12 (19.4)
Rhinorrhea	27 (43.5)
Miosis	3 (4.8)
Ptosis	9 (14.5)
Blepharedema	12 (19.4)
Facial edema	7 (11.3)
Facial sweating	17 (27.4)
Restlessness	20 (25.3)
**Additional features**	
Nausea	37 (46.8)
Vomiting	24 (30.4)
Photophobia	35 (44.3)
Phonophobia	36 (45.6)
**Laterality of pain**	
Right sided	33 (41.3)
Left sided	27 (33.8)
Side shift	20 (25.0)
Pain intensity	7.5 ± 2.3
Visual aura	1 (1.3)

*SD, standard deviation*.

Table 3 lists the periodicity of CH. In most patients, CH lasted for <2 weeks (41.8%), followed by 1 to 3 months (26.9%), 2 weeks to 1 month (23.9%), and >3 months (7.5%). The number of cluster periods since birth was <5 among 42 (55.3%) patients and 6–10 or >10 in 17 (22.4%) patients. The cluster periods lasted <6 months for 25 (45.5%) patients, 7–12 months for 12 (21.8%) patients, 1–2 years for 11 (20%) patients, and >2 years for 7 (12.7%) patients. Moreover, we reported the data on triggers of CH attacks in Supplementary Table 1.

**Table 3:** Periodicity of cluster headache.

**Variable**	**Frequency (percentage) or mean ± SD**
**Duration of cluster period (*****n*** **= 67)**	
<2 weeks	28 (41.8)
2 weeks−1 month	16 (23.9)
1–3 months	18 (26.9)
>3 months	5 (7.5)
**Number of cluster periods since birth (*****n*** **=** **76)**	
≤5	42 (55.3)
6–10	17 (22.4)
>10	17 (22.4)
**Duration between cluster periods (*****n*** **=** **55)**	
≤6 months	25 (45.5)
7–12 months	12 (21.8)
1–2 years	11 (20.0)
>2 years	7 (12.7)

*SD, standard deviation*.

**Figure 1:**
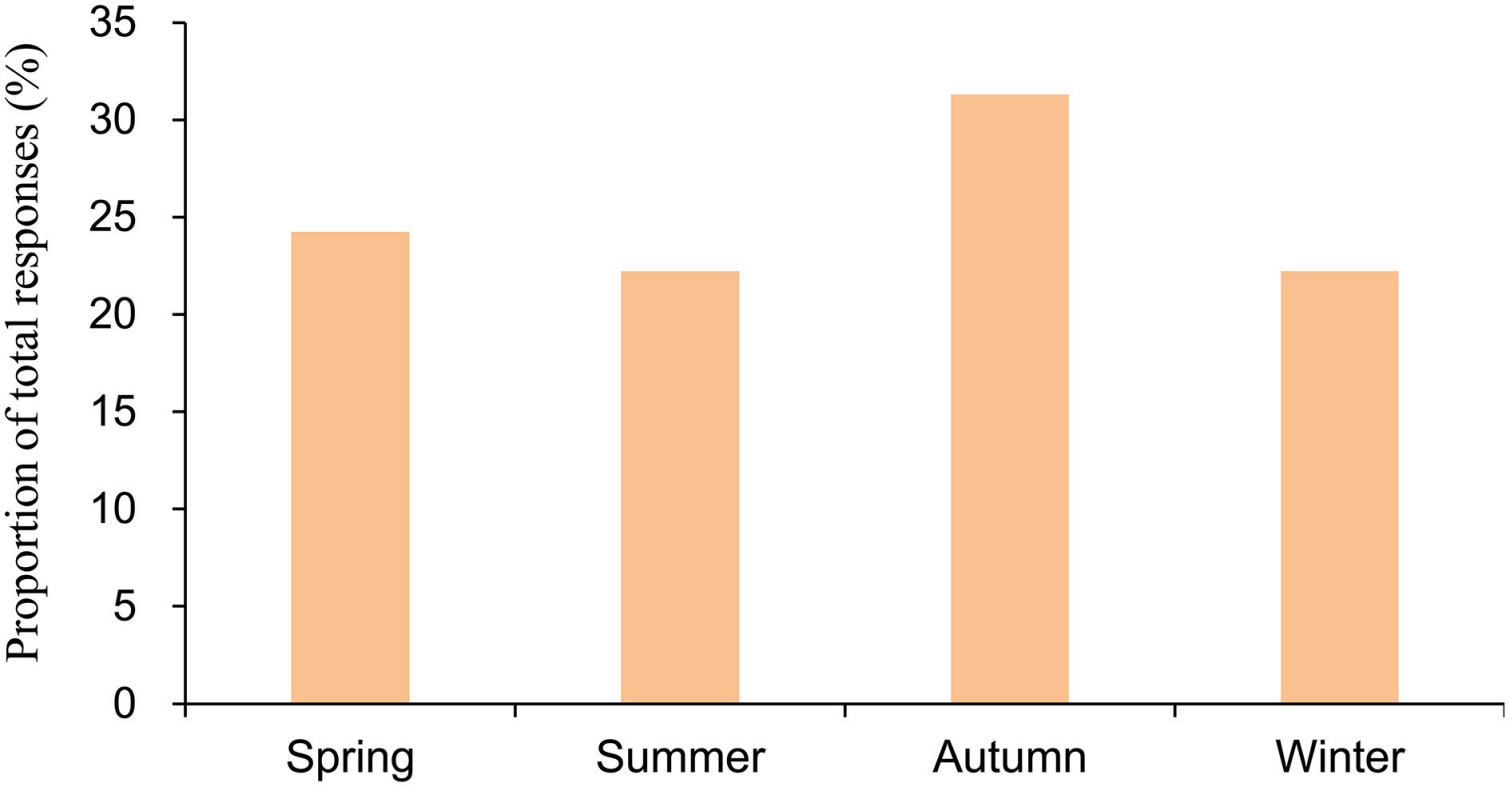
Season of onset of attacks among the patients with cluster headache. The numerator of the season in the bar consisted of the total number of responses in the 3 months. The denominator for the season is the total number of responses for all the months.

**Figure 2:**
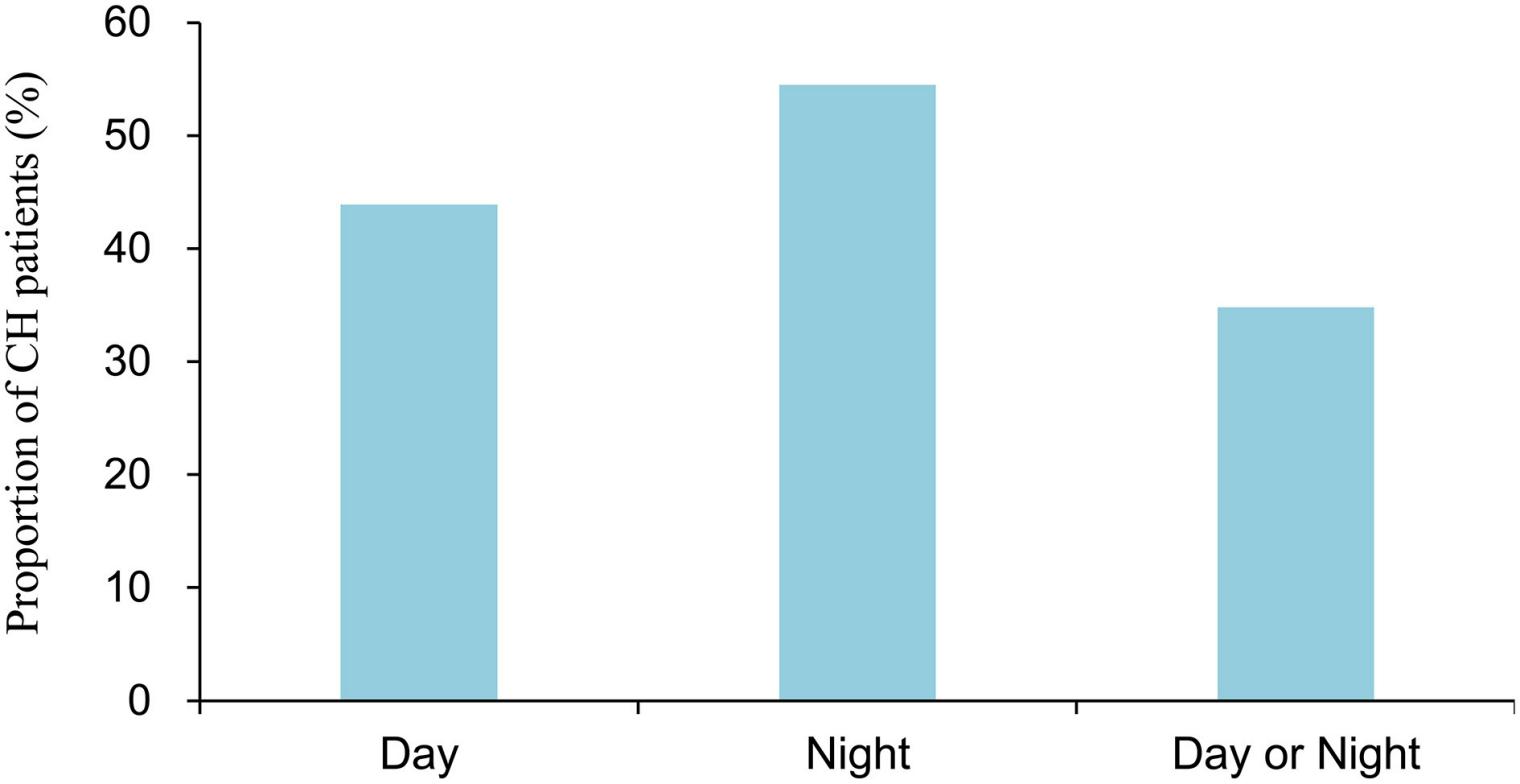
The frequency of cluster attacks at different times of the day.

Figure 1 shows the season specificity of CH. Generally, the distribution of CH was fairly balanced across seasons; the prevalence of CH was 24.2, 22.2, 31.3, and 22.2% in spring, summer, autumn, and winter, respectively, in Taiwan. Figure 2 illustrates the periodicity of CH; 54.5% attacks at night, 43.9% attacks during the day, and 34.8% attacks occurring at day or night.

## Discussion

Over a period of 4 years, we collected data from 80 CH patients who visited the outpatient department of a medical center (Tri-Service General Hospital) in Taiwan. The prevalence of the chronic CH subtype in Taiwanese CH patients in the present study is lower than that of Western CH patients (6, 7). We also demonstrated a lower percentage of positive family history of CH in our study sample compared to a positive family history rate from 5 to 17% in Western patients (1, 3, 6, 8). In addition, the percentage of occurrence of aura preceding CH was lower in our study sample than that in Western populations (14–23%) (3, 6, 8, 9).

The clinical characteristics of our study sample were comparable to those reported in Western CH studies; there was a higher male-to-female ratio, there was a similar age of onset of CH (in the third decade), pain mostly occurred in the temporal or retro-orbital regions and was more frequently right-sided than left-sided, and lacrimation was the most frequent cranial autonomic symptom (1, 3, 6, 8, 10–12). Although the rate of those previously regarded as migraine-specific accompanying symptoms was higher than that of autonomic symptoms, previous studies have reported that 54–78% of patients with CH experienced photophobia, 27–53% experienced nausea, 15–49% experienced phonophobia, and 12–32% experienced vomiting; these results are consistent with the results on our CH patients (11, 13). The high prevalence of smokers in our CH group is similar to the findings of Manzoni (14), who reported that ~90% of men and 70% of women CH patients were smokers. However, there was a higher proportion of positive history of smoking that ranged from 73 to 81.9% and from 53.3 to 55.0% in Western and Eastern CH populations, respectively; these are consistent with the present study results (3, 12, 14, 15).

However, there were several dissimilarities between the clinical characteristics of our study sample and those of Western CH patient populations. First, a greater male predilection was observed in our sample (male:female ratio, 5:1); the male-to-female ratios in Western studies range from 1.3 to 3.5:1 (9, 16). Previous studies also demonstrated that the diagnosis of CH was more difficult in women than in men until they are attended to in a specialized headache center and misdiagnosis was more prevalent in women (17–19). In addition, the recruitment of patients from a tertiary hospital possibly resulted in participation bias. Nevertheless, previous studies highlight a time-related reduction in male predominance among CH patients (13, 20). A possible explanation could be an improvement in the diagnostic accuracy, with more accurate CH diagnosis in women among Western populations. Another possible explanation could be the profound changes that has led to the redistribution among sexes in the last decades; the subsequent environmental and habitual factors, such as stress, alcohol consumption, and smoking, might play an important role (18, 19).

Second, we found a low prevalence (5%) of chronic CH in our study sample. Most of the patients in our study reported having episodic CH (95%), whereas only two (5%) patients had chronic CH. In Western populations, the prevalence of chronic CH among CH patients is ~15–26% (6, 7). However, a previous research conducted in Taiwan reported the absence of chronic CH (21) while studies of other Asian populations including Japan (3.5%) and China (7.5%) also reported a lower prevalence of chronic CH than that in Western populations (15, 22). Therefore, the prevalence of chronic CH may be relatively low in Asian patients compared to that in Western patients. Large-scale studies are warranted to determine if chronic CH is indeed less prevalent in Asian populations.

Third, CH is an inherited disorder and has multiple hereditary patterns; the most common pattern being autosomal dominant, followed by autosomal recessive (23). Studies in the Western population show a higher positive family history of CH (5–17%) (1, 3, 6, 8) compared to that of studies in Asian populations (0–6.7%) (12, 15, 21). Our findings corroborate the abovementioned results. Larger cohort samples of CH patients are needed to conclude whether there is indeed a lower percentage of reported CH among the patients' parents or siblings.

Fourth, occurrence of aura preceding a CH is more common in Western populations (14–23%), with visual aura being the predominant type (3, 6, 8, 9). In addition, Rozen and Fishman showed that 22% of the male and 19% of the female CH patients experience auras with no significant inter-sex differences (24). A previous study indicated that cortical spreading depression (CSD), which refers to a wave of neuronal hyperactivity followed by a wave of inhibition, was the most likely pathophysiological mechanism related to the generation of migraine auras (25). Moreover, CSD is assumed to be the cause of aura in CH, as with migraines (26). In our CH patients, the ratio of aura occurrence (~1%) was compatible with the results of the study in Asian populations (15, 21, 27) but lower than that of Western studies. This difference may be due to racial and genetic factors.

In Western studies, CH attacks are chiefly unilateral (right-sided preference); pain is localized in the orbital/periorbital region and occasionally radiates to the ipsilateral temple, jaw, maxilla, and upper teeth regions (3, 6, 8). In Eastern studies, CH patients predominantly experience pain in the temporal region and occurs with a higher frequency in the right side, as with the Western populations (12, 15, 21). Regarding side-shifting, 18–31% of CH patients had experienced headache on one side of the head during one bout and another side in a subsequent bout, which seemed to be likely in our CH group (3, 13). However, in our cohort, we noticed that the occipital region was involved more frequently (43.8%), in contrast to previous studies. Future research with larger cohort sizes is warranted, to address how frequently the occipital region is involved.

Previous studies in Western CH patients showed that 67.9–99.2% experience restlessness during attacks (3, 9, 11, 13). In contrast, Dong et al. found that a low frequency (38.3%) reported restlessness. This finding is compatible with the findings in other Asian populations, with 69.8% of CH patients from Japan (2) and 43.5% from Korea (27). In summary, this difference between Eastern and Western CH populations might be as a result of ethnic, social, and cultural factors.

In our study, the latency period was 7.8, 14.5, 7.7, 3.7, and 12.5 years in 2015, 2016, 2017, 2018, and 2019, respectively. The result of the linear trend was not significant, which indicates that the latency did not decrease over time. Although diagnostic delay was reduced significantly for each decade of CH onset, Frederiksen et al. revealed that there was associated prolonged latency in CH patients with migraine-like features and nocturnal attacks (17). However, the result is consistent with a previous study in Taiwan (21). The unexpected finding shows that physicians may not be vigilant enough in diagnosing CH. Therefore, further education about this devastating and treatable headache is important in the future.

The strength of this study is its well-controlled design. Moreover, CH was diagnosed by headache specialists according to a strict criterion using the Third edition of the International Classification of Headache Disorders (2). However, there were also several limitations. First, all the study participants were recruited from the neurology outpatient department of a tertiary hospital, which might limit the generalizability of the findings to other populations. Second, the cohort sample was relatively small. However, the precise diagnostic criteria could offset this drawback. Further studies with larger samples are needed to examine the similarities and differences in clinical features of CH between Asian and Western populations, especially in comparison with those of the Taiwan CH patient group.

In conclusion, the clinical characteristics of our CH patients differed from those of Western populations in several aspects including the lower prevalence of chronic CH, percentages of positive family history of CH, and occurrence of aura.

## Data Availability Statement

The original contributions presented in the study are included in the article/Supplementary Material, further inquiries can be directed to the corresponding author/s.

## Ethics Statement

The studies involving human participants were reviewed and approved by the Institutional Review Board of the Tri-Service General Hospital, National Defense Medical Center. Patient consent was obtained from all individual participants in this study. The patients/participants provided their written informed consent to participate in this study.

## Author Contributions

F-CY had full access to all the data in the study and took responsibility for its integrity. C-AK, G-YL, C-HT, Y-FS, and F-CY contributed to the study concept and design, acquisition, and interpretation of data. J-TL, C-KT, C-LT, Y-KL, and T-HH contributed to the analysis of the data. C-AK and F-CY participated in drafting the manuscript. All authors contributed to the collection and execution of the study. All authors have read and approved the final version of the manuscript.

## Conflict of Interest

The authors declare that the research was conducted in the absence of any commercial or financial relationships that could be construed as a potential conflict of interest.

## Supplementary Material

Supplementary Figure 1 Flowchart of the study patients' inclusion and exclusion process.

Supplementary Table 1 Triggers of cluster headache attacks.
