# Supplementary figures and images for: Clinical Features of Cluster Headache: A Hospital-Based Study in Taiwan
Source: Front Neurol. 2021 Apr 7;12:636888. doi: 10.3389/fneur.2021.636888 (PMC8058180; doi:10.3389/fneur.2021.636888)

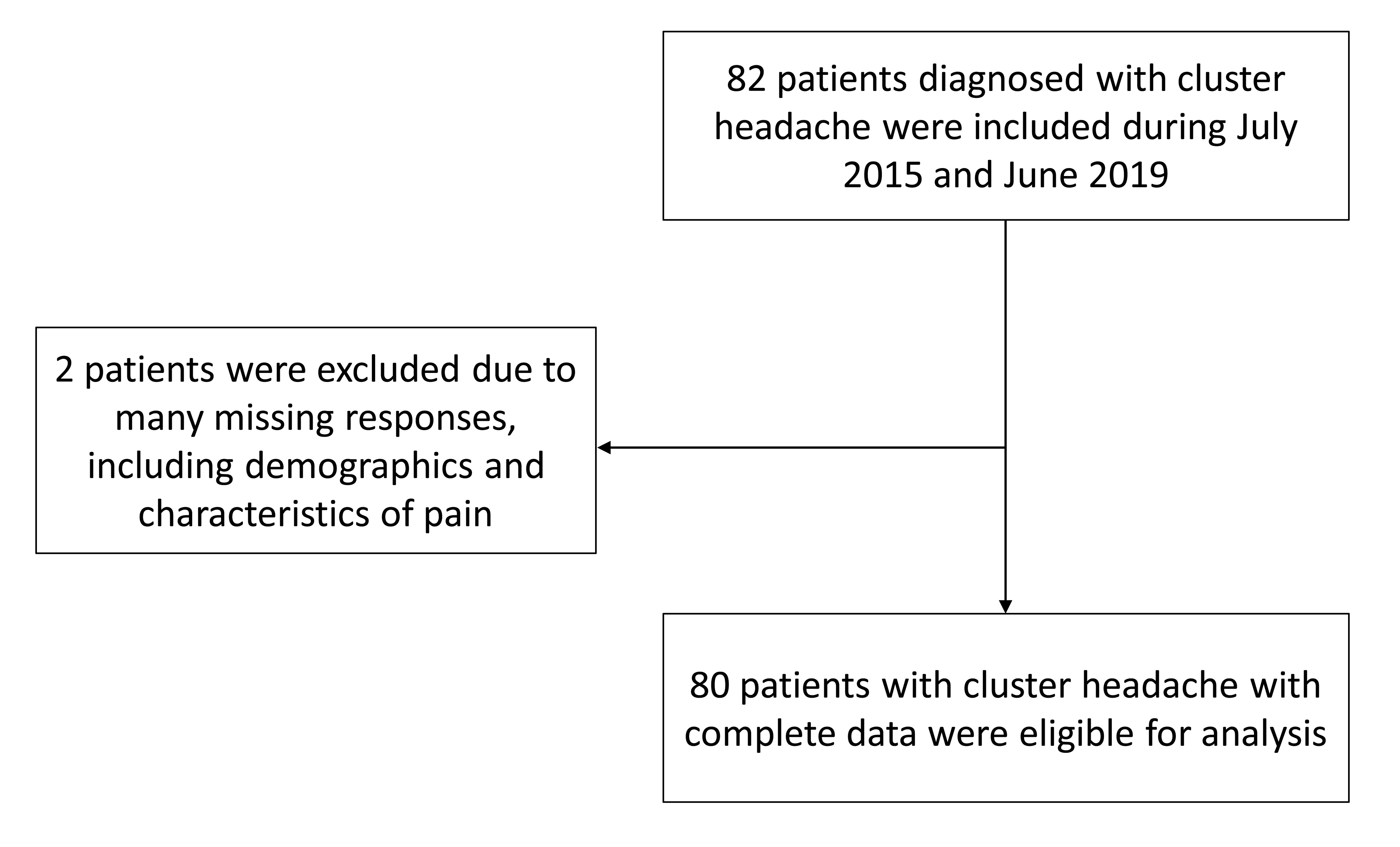

Supplement: Supplementary Figure 1 — Flowchart of the study patients' inclusion and exclusion process. [file Image_1.TIF]
