# Supplementary material for: Clinical Features of Cluster Headache: A Hospital-Based Study in Taiwan
Source: Front Neurol. 2021 Apr 7;12:636888. doi: 10.3389/fneur.2021.636888 (PMC8058180; doi:10.3389/fneur.2021.636888)
Supplement: Supplementary Table 1 — Triggers of cluster headache attacks. [file Table_1.doc]

**Supplemental Table 1.** Triggers of cluster headache attacks

| Category / Item | Frequency (%) |
| --- | --- |
| Smoking | 2 (2.5) |
| Foods |  |
| Cheese or Yogurt | 0 (0.0) |
| Hot dog | 1 (1.3) |
| Ice | 4 (5.0) |
| Chocolate | 2 (2.5) |
| Beverages |  |
| Beer | 8 (10.0) |
| White wine | 2 (2.5) |
| Red wine | 7 (8.8) |
| Champagne | 2 (2.5) |
| Other alcoholic beverages | 8 (10.0) |
| Coffee | 2 (2.5) |
| Tea | 1 (1.3) |
| Coke | 0 (0.0) |
| Characteristic odors |  |
| Cigarette or cigar | 1 (1.3) |
| Perfume | 1 (1.3) |
| Cleaning liquid or ammonia | 2 (2.5) |
| Situations |  |
| Lack of sleep or insomnia | 23 (28.8) |
| Tired or exhausted | 18 (22.5) |
| Eyestrain | 16 (20.0) |
| Pressure | 15 (18.8) |
| Depressive mood | 6 (7.5) |
| Anxiety | 11 (13.8) |
| Hunger | 1 (1.3) |
| Sex | 0 (0.0) |
| Relaxation | 2 (2.5) |
| Too much sleep | 6 (7.5) |
| Travel | 0 (0.0) |
| Holiday or weekend | 1 (1.3) |
| Weather change | 9 (11.3) |
| Humidity changes | 4 (5.0) |
| Mountaineering | 1 (1.3) |
| Intense exercise | 4 (5.0) |
| Cold wind | 8 (10.0) |
| With hat (tightly) | 3 (3.8) |
| Confined space (not well ventilated) | 8 (10.0) |
| Take a plane | 0 (0.0) |
| Cold spell | 9 (11.3) |
